# Repetitive Negative Thinking and Eating Disorders: A Meta-Analysis of the Role of Worry and Rumination

**DOI:** 10.3390/jcm10112448

**Published:** 2021-05-31

**Authors:** Sara Palmieri, Giovanni Mansueto, Simona Scaini, Gabriele Caselli, Walter Sapuppo, Marcantonio M. Spada, Sandra Sassaroli, Giovanni Maria Ruggiero

**Affiliations:** 1 Division of Psychology, London South Bank University, London SE1 0AA, UK; s.palmieri@milano-sfu.it (S.P.); g.caselli@milano-sfu.it (G.C.); w.sapuppo@milano-sfu.it (W.S.); spadam@lsbu.ac.uk (M.M.S.); 2 Studi Cognitivi, Cognitive Psychotherapy School and Research Center, 20132 Milan, Italy; g.mansueto@milano-sfu.it (G.M.); s.sassaroli@milano-sfu.it (S.S.); 3 Department of Psychology, Sigmund Freud University, 20143 Milan, Italy; s.scaini@milano-sfu.it; 4 Department of Health Sciences, University of Florence, 50121 Florence, Italy

**Keywords:** repetitive negative thinking, rumination, worry, eating disorder, anorexia, bulimia, binge eating disorder

## Abstract

The role of worry and rumination in eating disorders (EDs) is controversial. This meta-analysis of the literature is aimed at clarifying the relationship between repetitive negative thinking (RNT) and EDs. In accordance with the PRISMA criteria, a comprehensive search of the literature was conducted on PubMed and PsycInfo from inception to March 2021. Search terms: “eating disorder/anorexia/bulimia/binge eating disorder” AND “worry/rumination/brooding/repetitive thinking”. A manual search of reference lists was also run. Forty-three studies were included. RNT was found to be associated with anorexia, bulimia, and binge eating disorder. A moderating effect was found for “presence/absence ED diagnosis” and “subtype of ED symptom”. ED patients showed higher RNT than the general population. No differences were observed for age or between worry and rumination in the magnitude of their association with EDs.

## 1. Introduction

Repetitive negative thinking (RNT) is a cognitive process characterised by a repetitive, frequent, and self-focused form of thinking [1]. Worry and rumination have been grouped under the construct of RNT [2,3]. Worry has been defined as a chain of thoughts and images laden with negative affects and relatively uncontrollable [4]. Worry is an attempt to engage in mental problem-solving on an issue whose outcome is unknown but contains the possibility of being negative. Rumination is defined as thoughts that repetitively focus attention on negative emotions and symptoms, their causes, meanings, and consequences [5]. Rumination can take verbal and imaginary forms [6], characterized by the tendency to repeatedly think on the self, upsetting events, and personal concerns [7]. Worry is usually focused on problem-solving and is more future-oriented, whereas rumination often consists of themes of loss and typically has a focus on past problems [8].

An extensive literature base has suggested that both worry and rumination are cognitive processes present across diverse disorders [2]. Worry is associated with anxiety disorders [9,10,11,12] and major depressive disorder [13,14,15]. Rumination is associated with both the development and persistence of mood and anxiety disorders [16,17,18,19,20,21,22,23,24], addictive behaviours [25,26], and schizophrenia [27].

It is extensively acknowledged that preoccupation with the control of eating, weight, and shape is conceptualized as core feature of eating disorder (ED) psychopathology [28,29]. Thus, it is conceivable that individuals presenting with EDs may report a stronger tendency to engage in RNT. A recent meta-analysis by Smith, Mason and Lavender [30] pointed out that rumination is concurrently and prospectively associated with ED psychopathology and that individuals with EDs showed higher levels of rumination than those without an ED. Although the association between rumination and EDs has been explored, no study has systematically examined the role of worry in EDs. Notwithstanding some evidence showing raised levels of worry in patients with EDs compared to controls from the general population [31,32,33], it is not possible to draw a conclusion about the association between worry and EDs due to the lack of a systematic review or meta-analysis.

Thus, an increasing number of studies have explored the association between RNT and EDs, and the relationship between rumination and EDs has been highlighted in the meta-analysis by Smith and colleagues [30]. However, to date, no qualitative or quantitative reviews have been performed that take into account both worry and rumination and possible moderators of the relationship between RNT and EDs. Clarifying this relationship could have implications for clinical practice, especially with respect to interventions aimed at interrupting RNT.

Through the use of meta-analytic techniques, the present study aimed to present a comprehensive evaluation of the literature on EDs, worry and rumination in order to: (1) extend the literature and estimate the magnitude of the association between EDs, worry and rumination; (2) explore the role of some moderators such (a) “subtypes of ED symptoms”, the (b) “presence vs. absence of a diagnosis of ED” and (c) “worry vs. rumination” and (3) “mean age of the sample” in shaping heterogeneity.

## 2. Materials and Methods

### 2.1. Study Selection

The methodology of the study selection will be reported in accordance with the Preferred Reporting Items for Systematic Reviews and Meta-Analyses (PRISMA) guidelines [34].

#### 2.1.1. Eligible Studies Included

The inclusion criteria applied to the literature search were: (a) English language articles published in peer-reviewed journals; (b) diagnosis of ED determined according to the standardized diagnostic criteria, including the Diagnostic and Statistical Manual of Mental Disorders (DSM) from the third to the fifth edition [35,36,37,38,39], the Research Diagnostic Criteria (RDC) [40] or the International Classification of Diseases (ICD) from the sixth to the tenth edition [41,42,43,44,45] and assessing worry and/or rumination; (c) studies using a case–control design/prospective cohort studies/large population-based cross-sectional studies/experimental studies and (d) information available to determine the effect size.

Studies including participants with a diagnosis of neurological and/or neurocognitive organic impairment, or co-occurrent psychiatric disorders or obese participants, were not included, as well as studies on cognitive processes not specifically referring to worry and rumination.

#### 2.1.2. Information Sources and Search

PubMed and PsycInfo were systematically searched from inception to 31 March 2021. Furthermore, a manual search of reference lists from all the articles selected, full text-reviews and significant reviews was run. Search terms included: eating disorder/anorexia/bulimia/binge eating disorder combined using Boolean “AND” operator with worry/rumination/brooding/repetitive thinking.

#### 2.1.3. Study Selection, Data Collection Process and Data Items

The studies’ eligibility was assessed through the following procedure: title screening, abstract screening and full paper screening. Titles and abstracts were screened by S.P. Articles appearing potentially relevant were retrieved by S.P. and independently assessed by S.P. and G.M. Consistent with the previous studies [46,47,48], disagreements on eligibility were resolved by consensus among authors (intercoder reliability: Cohen’s Kappa coefficient = 0.70). The following assumptions were made: if not specified, participants were considered without co-occurrent psychiatric disorders or neurological or neurocognitive organic impairment.

#### 2.1.4. Assessment of Risk of Bias in Individual Studies

In order to ascertain the validity of the eligible studies, two investigators independently rated each study on the basis of the following markers: homogeneity of the sample regarding the diagnosis if present, appropriateness of random allocation if necessary and presence of a comparable group if appropriate. Disagreements were resolved by consensus [46,47,48] (intercoder reliability: Cohen’s Kappa coefficient = 0.80).

### 2.2. Quality Assessment

In accordance with previous studies [46,47,48,49], the quality of each eligible study was assessed independently by two investigators (S.P. and G.M.) using the Newcastle Ottawa scale for case–control studies and its adapted form for cross-sectional studies [49,50]. Disagreements were resolved by consensus (intercoder reliability: Cohen’s Kappa coefficient = 0.85).

### 2.3. Data Analyses

Analyses were conducted using Comprehensive Meta-analysis, CMA version 2.0 by Biostat. Using a random effects model, we calculated the effect size (ES), which was reported here as the standardized difference between the means of the two groups (Cohen’s d), together with their 95% CIs. According to Cohen’s criteria (Cohen, 1988), an ES of <0.20 is considered a small effect, an ES of about 0.50 is a moderate effect and ES of about 0.80 is a large effect. For the purposes of the current study, a positive ES indicated an association between RNT and eating problems. Each ES was calculated for each self-report symptoms scale included in the identified studies and averaged across measures to obtain an ES for each study [51,52].

In order to address the publication bias, i.e., the possibility that published studies have larger mean ES than unpublished studies, we checked the results using the “Trim and Fill” procedure [53] and the Classic Fail-safe Number method [54]. The “Trim and Fill” procedure is a nonparametric method that evaluates the effect of potential data censoring on the meta-analysis [53]. Using this method, a plot of each study’s ESs against the meta-sample’s ES and standard error was built. These plots should be shaped as a funnel when no data censoring is present. Since smaller or nonsignificant studies are less likely to be published, studies in the bottom left/right-hand corner of the plot are often omitted [55]. The most symmetrically right/left unmatching studies in the meta-analysis are thus trimmed and replaced with their missing counterparts imputed or “filled” in as mirror images of the trimmed outcomes [56]. This allows for the computation of an adjusted ES and relative CI. The Classic Fail-safe Number estimates the number of studies with nonsignificant findings, which are necessary to make the combined ES nonsignificant [56]. Meta-analyses with a fail-safe number higher than (5* studies number + 10) are usually considered free from publication bias [54].

The presence of heterogeneity across the studies was evaluated by the I^2^ index, which measures the proportion of total variation due to real differences in the variability of ESs among studies [56]. The Q statistic was used to test the heterogeneity of the specific set of ESs and the effects of the selected moderators [56]. We considered the following moderators: (a) subtypes of ED symptoms (anorexia nervosa (AN), bulimia nervosa (BN) and binge eating disorder (BED)); (b) presence vs. absence of any diagnosis of EDs (i.e., the comparison between patients and healthy controls from the population); (c) subtype of RNT: worry or rumination and (d) mean age of the sample.

The 1st and 2nd authors independently coded the qualitative moderators in each study, and they reached consensus in the case of disagreements. No disagreements were found among the authors.

For dichotomous moderators, we carried out a subgroup analysis based on a mixed-effect model, assuming a common among-study variance component across the subgroups and a random effect model to combine the subgroups. A Q-test was used to test for heterogeneity across the subgroups [56].

## 3. Results

### 3.1. Study Selection

**Figure 1:**
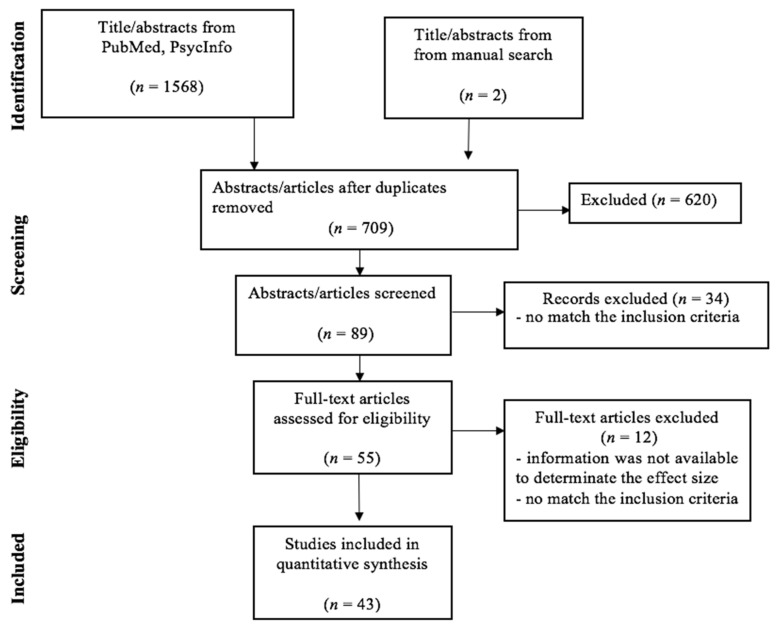
Identification of independent studies for inclusion in the meta-analysis (flow chart). Forty-three studies of 57 cohorts, because some studies were considered more than once. Search Strategy: Limits: English; Search terms included: worry/rumination/brooding/repetitive thinking combined using Boolean “AND” operator with eating disorder/anorexia/bulimia/binge eating disorder.

The search of the PubMed and PsycInfo databases and the manual search showed a total of 1570 citations. After removing the duplicates and reviewing the abstracts to exclude those that clearly did not meet the criteria (*n* = 620), 89 remained. Of these, 34 studies were excluded, because they did not meet the inclusion criteria. Of the 55 studies that remained, 12 studies were further discarded. Figure 1 illustrates the search and screening process. The 43 articles that met the inclusion criteria are listed in Table 1 and Table 2, along with their study characteristics. The total sample size of the selected studies comprised 10,391 participants; among them, 1345 were ED cases (AN = 521, BN = 278, BED = 325 and EDNOS = 221), and 9046 were healthy controls from the general population. The following is a summary of the literature concerning the association between ED, worry and rumination.

A total of 43 studies, 10 on worry and 35 on rumination, were identified for inclusion in the meta-analysis. Thirteen reports were considered more than one time, since some studies included both AN and BN samples [32,33,57,58,59,60,61] or included clinical and general populations [62,63,64] or included different kinds of general populations [65] or included both worry and rumination [57,66], leaving a total of 57 entries for the meta-analysis.

### 3.2. Study Quality

None of the studies fulfilled all the Newcastle-Ottawa quality criteria. In twelve case–control studies, six studies scored 7/10, three studies scored equal to 6 and three studies scored 5/10. In thirty-three cross-sectional studies, three studies scored 8/10, 26 studies scored equal to 7, three study scored 6/10 and one scored 5/10. More details about the study quality for the case–control and cohort studies are reported in the Supplementary Materials in Tables S1 and S2, respectively.

### 3.3. RT and Eating Problems

**Figure 2:**
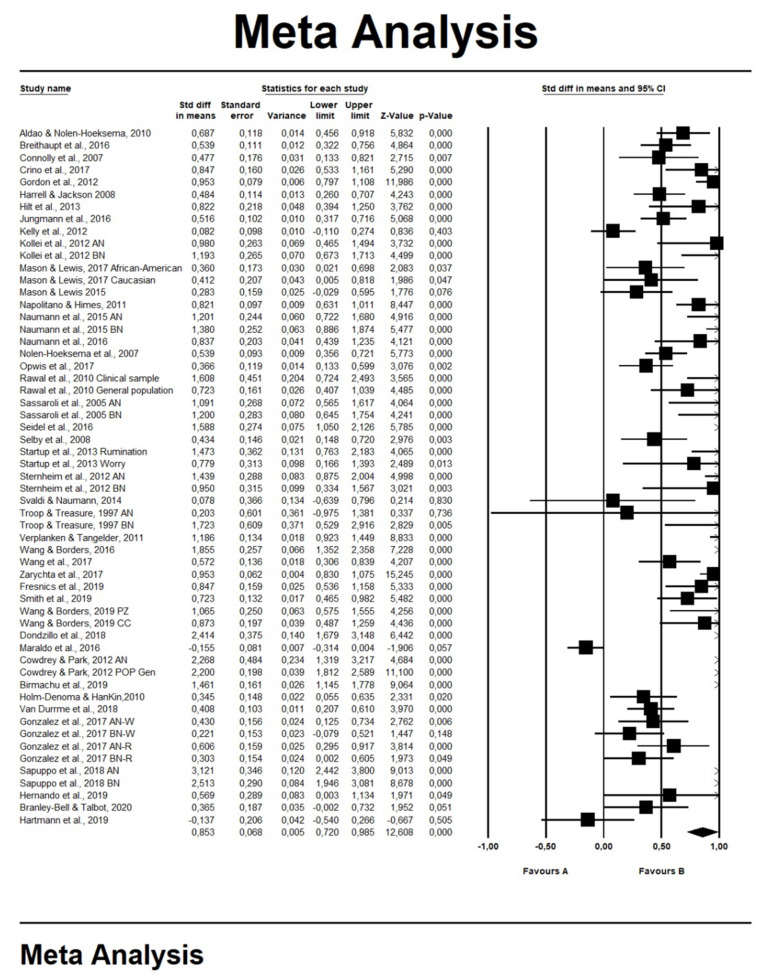
Forest plots for repetitive thinking and eating behavior. Box indicates effect sizes (ESs), while error bars represent 95% confidence intervals (CIs). The rumble represents the average ES. Note: Favors A = negative association between repetitive thinking and eating disorders. Favors B = positive association between repetitive thinking and eating disorders. The rumble represents the average ES.

The 57 entries selected showed an average ES of 0.85 (SE: 0.07, 95% CI: 0.72–0.98; Test of Null (2-Tail): *z*-value = 12.61, *p* < 0.001, *k* = 57), which indicated a large positive association between eating problems and RNT (Figure 2). The ES is a global measure and has been calculated in all the studies included in the quantitative analysis. Duval and Tweedie’s trim-and-fill procedure indicated only five missing studies (adjusted ES *g* = 0.90, 95% CI: 0.75–1.06), with a classic fail-safe number estimated at 6897. Since we found heterogeneity among the studies (Q = 559.79, *p* < 0.001, I^2^ = 90.000), we conducted further analyses by testing the roles of the possible moderators.

#### Moderator Analyses: Subtypes of ED Symptoms, Presence vs. Absence of Diagnosis of EDs, Worry–Rumination and Age

For each moderator, a separate model was tested. For more details, see Table 3. The subgroup analysis revealed differences in ES for the types of ED symptoms (Q = 11.65, *df* = 2, *p* = 0.003): AN *d* = 1.35 (SE:0.20, 95% CI: 0.96–1.74; Test of Null (2-Tail): *z*-value = 7.76, *p* < 0.001, *k* = 14), BN *d* = 0.75 (SE:0.11, 95% CI: 0.54–0.97; Test of Null (2-Tail): *z*-value = 6.80, *p* < 0.001, *k* = 14); BED, *d* = 0.50 (SE:0.15, 95% CI: 0.20–0.80; Test of Null (2-Tail): *z*-value = 3.29, *p* = 0.001, *k* = 7).

We also found differences in the ES between studies based on clinical samples vs. studies based on the healthy controls from the general population (Q = 8.15, *df* = 1, *p* = 0.004): clinical samples ES: *d* = 1.14 (SE: 0.13, 95% CI: 0.87–1.40; Test of Null (2-Tail): *z*-value = 8.44, *p* < 0.001, *k* = 24); general population ES: *d* = 0.69 (SE:0.08, 95% CI: 0.54–0.85; Test of Null (2-Tail): *z*-value = 8.97, *p* < 0.001, *k* = 33).

No differences emerged from the comparison between the studies on worry vs. studies on rumination (Q = 2.45, df = 1, *p* = 0.118): worry ES: *d* = 1.04 (SE: 0.14, 95% CI: 0.76–1.32, Test of Null 2-Tail: *z*-value = 7.28, *p* < 0.001, *k* = 15); rumination ES: *d* = 0.79 (SE: 0.08, 95% CI: 0.64–0.94, Test of Null 2-Tail: *z*-value = 10.36, *p* < 0.001, *k* = 42).

Finally, there was not a significant effect of the variable “age” in shaping heterogeneity (age: β point estimate = −0.0108, SE = 0.009, 95% CI: −0.03–0.01, *z*-value = −1.12, *p* = 0.26, *k* = 55).

## 4. Discussion

To our knowledge, this is the first study on the relationship between EDs, worry and rumination based on a meta-analytic methodology. The main findings of the present meta-analysis indicate a significant association between RNT and EDs, given that: (a) RNT is highly associated with eating problems in both clinical and nonclinical samples (ESs range from 0.50 to 1.35) and (b) ED patients show higher levels of RNT than the general population. These findings are consistent with Smith and colleagues’ findings [30] but differ from these given that both worry and rumination have been found to be associated with ED symptoms. Moreover, our findings suggest that the strength of the association is not influenced by the age of the participants.

These findings raise an important question: How does RNT play a role in ED symptoms, such as dieting or binge eating? It has been shown that negative beliefs and negative emotions might act as a trigger for RNT that, in turn, further maintains the experience of emotional distress [2,3,66,97,98,99]. Moreover, it is well-known that dieting and binge eating could be a coping strategy to tackle negative emotions [29,99,100]. Should the latter perspective be taken, dieting could be construed as a strategy to cope with negative thoughts and/or emotions that act as a trigger of RNT. Binge eating could be a behavior aimed to reduce chronic stress due to RNT focused on dieting or independently from it. Even though clinical models, such as the Self-Regulatory Executive Function (S-REF) model [101], the Emotional Cascade Model (ECM) [73] or Fairburn’s model [29], might partially support these assumptions, further studies are required to directly test these hypotheses.

The Self-Regulatory Executive Function model (S-REF model) [101] postulates that several maladaptive forms of coping, including repetitive negative thinking (desire thinking, rumination and worry), maintain psychological distress. These maladaptive forms of coping are termed the “Cognitive Attentional Syndrome” (CAS) [102], which is activated and maintained by metacognitive beliefs (i.e., information individuals hold about their own cognition and coping) [102]. CAS is problematic, because it causes negative cognitive–affective states to remain in the consciousness rather than spontaneously decay, leading to failures to modify self-beliefs about control over the mind [103]. Given the association observed between RNT and ED symptoms, and the association between metacognitive beliefs and both EDs and behaviors [48], the S-REF model could explain the role of RNT in ED symptoms.

As postulated by the emotional cascade model [73], an event that triggers a negative emotion may lead to rumination about the event, increasing the intensity of the negative emotions. Furthermore, stress and negative emotional states may increase the level of rumination, which, in turn, may lead to an escalation (cascade) of negative feelings [104]. As a result, an individual may engage in eating behaviors as a coping strategy to tackle negative mood states. It may be assumed that worry and negative emotion are related to each other in the same way. There is mounting evidence that worry may maintain the experience of emotional distress [98].

As regards the Fairburn’s “transdiagnostic” model of ED [29], it was proposed that binge eating could be triggered, among other factors, by adverse events and negative mood states. In turn, binge eating will tend to improve, albeit temporarily, a negative mood and serve as a distraction from negative thinking patterns. It can therefore be argued that an ED mindset characterized by cognitive processes such as worry and rumination exist, and this is activated in the presence of adverse events, leading to a negative mood and the maintenance of ED behaviors. These behaviors may serve to decrease negative emotions, interrupt worry and rumination and help manage (in the short-term) the adverse event. However, the manner in which worry, rumination and negative affectivity may interact in EDs remains unclear, with further studies required to disentangle this complex relationship.

The findings of the current meta-analysis suggest that RNT is associated with all subtypes of ED symptoms supporting a vision of RNT as a transdiagnostic process [2]; nevertheless, some differences may be identified in the strength of the association between RNT and the subtypes of ED symptoms: based on the ES values, the association between RNT and the subtypes of ED symptoms might be stronger in AN than in BN and BED. Taking as a framework the S-REF model [101], it could be hypothesized that there are differences among the ED symptom subtypes in maladaptive metacognitive beliefs that activate and maintain maladaptive forms of coping such as RNT; as highlighted in a recent systematic review [48], maladaptive metacognitive beliefs appear to be stronger in AN than in other ED subtypes. Furthermore, the moderate ES for BED could suggest that BED symptoms could be more closely related with a different form of RNT process such as desire thinking [78,105] rather than worry and rumination, albeit the scarce number of published studies on BED suggests caution in this interpretation.

Furthermore, our data, differently from a previous meta-analysis that exclusively focused on rumination [30], suggests that there is no difference between worry and rumination in the relationship with eating problems. This suggests, in line with the construct of RNT [2,3], that both processes are implicated in eating problems. Notwithstanding this observation, the additional data on worry may be in line with some evidence suggesting that, beyond having a common characteristic in repetitive thought, rumination and worry may have differential effects on the severity of mental illness [106,107]. Compared to rumination, worry appears to be a more influential cognitive vulnerability factor in predicting the increasing symptoms over time [106]. Focusing on the ED symptoms, negative thoughts about the weight and shape could activate worrying about eating and weight gain as a control strategy and associated negative beliefs and emotions; moreover, the worry about food may be a distraction from the preoccupations regarding self-esteem and interpersonal relations [32]. However, people with ED symptoms might also be worried about factors not strictly connected to the core features of EDs.

Finally, our data showed that age does not moderate the relationship between RNT and eating problems, leading to the hypothesis that the vicious circle among RNT, negative beliefs and emotions and eating behaviors could be independent from the passing of time. However, our data does not allow for further speculations on this issue.

A number of clinical and research implications rose from the findings of the current meta-analysis. Firstly, the assessment of RNT, in terms of worry and rumination, should not be overlooked during the anamnesis of a patient presenting with eating problems. Secondly, it could be important to inform patients presenting with eating problems that RNT is a disadvantageous mechanism that leads to worse clinical outcomes [108]. Thirdly, a treatment aimed to decrease the propensity to engage in RNT [108], such as Metacognitive Therapy [12] and Rumination-Based Therapy [109], should be considered as treatment options for EDs. Moreover, and based on the observed relationship between RNT and eating problems in the general population (ES = 0.69), early intervention in tackling RNT may help prevent more severe forms of problematic eating behaviour. Future research on EDs and eating problems could explore in depth the role of worry and rumination.

The value of this meta-analysis should be interpreted considering the strengths and limitations of the included studies. A significant strength is that the investigation of the relationship between worry, rumination and EDs was conducted across different subtypes of EDs, rather than in a specific ED, emphasizing the role of RNT in EDs. Some limitations should be also considered. The instruments used in the reviewed studies to evaluate the worry and rumination are not homogeneous: some studies used an instrument specific for ED (Ruminative Response Scale for Eating Disorders, RRS-ED); others used generic instruments (Ruminative Response Scale, RRS; Penn State Worry Questionnaire, PSWQ) or carried out an experiment. The majority of the studies were retrospective; hence, they are subject to a possible recall bias. Furthermore, the sample sizes were often small and composed only of female participants. Moderator analyses could be affected by this specific limitation. Thus, the analyses on moderators should be considered to be exploratory in nature. Moreover, focusing exclusively on published studies entails that the information about negative results is likely to be lost [110].

## 5. Conclusions

In conclusion, RNT represents a transdiagnostic phenomenon also involved in EDs. Future directions for research should include studies that: (1) explore in depth the worry and rumination in BED, since most studies have focused on AN and BN; (2) evaluate the relationship between RNT and EDs considering the possible confounder variables such as anxiety and depression [63,111]; (3) explore the relationship between RNT and negative emotion in EDs and (4) include longitudinal designs.

## Figures and Tables

**Table 1:** Summary of the demographic characteristics of the studies assessed and the relationship between worry and eating behavior.

Source	Study Design	Sample-Size	Age (Years) Mean ± SD	Sex % (*n*)	Diagnostic Tool Eating Measure	Sub-Types of ED Symptoms	Worry Measure
Napolitano and Himes 2011 [31]	case–control	cases: 46 binge eating group without BED: 186	-	F: 100% (232)	DSM-IV-TR EDDS	BED	FOBES
Sassaroli et al. 2005 [32]	case–control	cases: 63 controls: 30	23.06 ± 4.54 vs. 26.32 ± 4.27	F: 100% (93)	SCID-I	AN BN	PSWQ
Sternheim et al. 2012 [33]	case–control	cases: 45 controls: 37	25.8 ± 8.5 vs. 27.8 ± 10.2	not reported	DSM-IV EDE	AN BN	PSWQ Catastrophizing Interview
González et al. 2017 [57]	cross-sectional	general population: 176	31.2 ± 13.3	F: 67% (118)	EAT	AN BN	PSWQ
Kollei, et al. 2012 [58]	case–control	cases: 66 control: 33	AN: 26.94 ± 9.15 BN: 25.94 ± 8.25 CG: 26.91 ± 8.48	F: 95.45% (63) vs. 69.7% (23)	DSM-IV	AN BN	CITQ
Sapuppo et al. 2018 [60]	case–control	cases: 84 controls: 38	23.39 ± 4.75 vs. 25.31 ± 5.4	F: 100% (122)	SCID-I	AN BN	PSWQ
Startup et al. 2013 [66]	cross-sectional	cases: 62	26.6 ± 7.8	F: 93.5% (58)	DSM-IV EDE	AN	PSWQ
Zarychta et al. 2017 [67]	cross-sectional	general population: 1260	16.38 ± 0.80	F: 41.7% (525)	MBSRQ	-	MBSRQ
Crino et al. 2019 [68]	case–control	cases: 90 controls: 97	25.23 ± 8.33 vs. 20.63 ± 6.37	F: 100% (187)	DSM-5	AN BN BED No-ED	TCQ
Hartmann et al. 2019 [69]	cross-sectional	cases: 95	AN: 23.64 ± 0.62 BN: 26.09 ± 1.17	F: 98%(49) F: 97.78%(44)	EDE-Q	AN BN	Self-constructed worry item

Note: AN: Anorexia Nervosa; BN: Bulimia Nervosa; BED: Binge Eating Disorder; CG: Control group; DSM-IV: Diagnostic and Statistical Manual of Mental Disorders; EAT: Eating Attitudes Test; EDE: Eating Disorder Examination; FOBES: Functional assessment of binge eating; PSWQ: Penn State Worry Questionnaire; CITQ: Control of Intrusive Thoughts Questionnaire; MBSRQ: The Multidimensional Body-Self Relations Questionnaire.

**Table 2:** Summary of the demographic characteristics of the studies assessed the relationship between rumination and eating behavior.

Source	Study Design	Sample-Size	Age (Years) Mean ± SD	Sex % (*n*)	Diagnostic Tool Eating Measure	Sub-Types of ED Symptoms	Rumination Measure
González et al. 2017 [57]	cross-sectional	general population: 176	31.2 ± 13.3	F: 67% (118)	EAT-26	AN BN	RRS
Naumann et al. 2015 [59]	experiment case-control	cases: 36 controls: 19	AN: 24.94 ± 8.92 BN: 23.28 ± 6.37 CG: 23.32 ± 8.02	F: 100% (111)	DSM-IV-TR EDE	AN BN	Rumination experimentally induced by Nolen-Hoeksema and Morrow’s task (1993) RSQ
Troop and Treasure 1997 [61]	case–control	cases: 21 controls: 15	AN: 23.3 ± 5 BN: 25.4 ± 10.8 CG: 29.5 ± 9	F: 100% (36)	ICD-10	AN BN	Coping Strategies Interview
Cowdrey and Park 2012 [62]	cross-sectional	general population: 228	24.03 ± 7.62	F:100% vs. 100%	EDE-Q	AN and Eating pathology	RRS-ED
		AN: 42	24 ± 8.31				
Rawal et al. 2010 [63]	Study 1 cross-sectional	students: 177	22.39 ± 5.13	F: 68.92% (122)	DSM-IV EDE-Q	AN	Study 1 & 2: RRS
	Study 2 case–control	cases: 13 controls: 13	26.38 ± 8.77 vs. 25.77 ± 4.85	F:100% vs. 100%	MINI EDE		
Wang and Borders 2018 [64]	Study 1 cross-sectional	undergraduate students: 126	19.7 ± 1.10	F: 84% (106)	EDE-Q	Eating pathology	RRS
	Study 2 cross-sectional	cases: 85	24.57 ± 9.95	F: 87.1% (74)			
Mason and Lewis 2017 [65]	cross-sectional	general population Caucasian: 100	20.14 ± 1.82	F = 100%	DSM-5 (binge eating episode)	-	RSQ
		African-America: 84	19.75 ± 1.86				
Startup et al. 2013 [66]	cross-sectional	cases: 62	26.6 ± 7.8	F: 93.5% (58)	DSM-IV EDE	AN	CERTS
Connolly et al. 2007 [70]	cross-sectional	general population: 140	19.5 ± 2.57	F 100% (140)	BES EDE-Q	BED	BARQ
Nolen-Hoeksema et al. 2007 [71]	cross-sectional	general population: 496	13.5 ± 0.67	F = 100%	DSM-IV EDE	BN	RSQ
Harrell et al. 2008 [72]	cross-sectional	general population: 329	19.31	F = 100%	Dieting and Bingeing Severity Scale	-	AFCI
Selby et al. 2008 [73]	cross-sectional	general population: 200	18.6 ± 2.36	F: 68.5% (137)	EDI	BN	CERQ
Aldao and Nolen-Hoeksema 2010 [74]	cross-sectional	undergraduate students: 252	18.44 ± 0.66	F: 55.6% (140)	EDE-Q	-	RRS
Holm-Denoma and Hankin 2010 [75]	cross-sectional	general population: 191	14.5 ± 1.4	F = 100%	EDDS	BN	CRSQ
Verplanken and Tangelder 2011 [76]	cross-sectional	students: 303	24 ± 4	F: 50.16% (152)	EDS-5	-	Negative Body Image Thinking
Gordon et al. 2012 [77]	cross-sectional	general population: 780	19.27 ± 2.12	F: 65.7% (512)	BES	BED	RRS
Kelly et al. 2012 [78]	cross-sectional	general population: 419	18.95 ± 1.33	F = 100%	EDE-Q	BED	CERQ
Hilt et al. 2013 [79]	cross-sectional	general population: 101	12.7 ± 1.14	F = 100%	Children’s Eating Attitudes Test ChEAT	Eating pathology	CRSQ
Svaldi and Naumann 2014 [80]	cross-sectional	cases: 30	46.33	F = 100%	DSM-IV-TR EDE	BED	PTQ
Mason and Lewis 2015 [81]	cross-sectional	general population: 164	-	F = 100%	BES	BED	CERQ
Breithaupt et al. 2016 [82]	cross-sectional	general population: 353	21.93 ± 5.78	F = 85% (300)	EAT-26	BN	RRS
Jungmann et al. 2016 [83]	cross-sectional	general population: 414	47.2 ± 16.7	F: 54% (223)	EDI-2	BN	RSQ
Maraldo et al. 2016 [84]	cross-sectional	community participants: 313 students: 296	34.74 ± 11.36 vs. 19.44 ± 1.75	F = 100%	EDE-Q	Eating pathology	RRS
Naumann et al. 2016 [85]	case-control	cases: AN: 42 BN: 40 controls: 41	AN: 25.71 ± 10.65 BN: 25.78 ± 8.49 CG: 25.61 ± 10.30	F = 100%	DSM-IV EDE	AN BN	Self-constructed Visual Analog Scales
Seidel et al. 2016 [86]	case-control	cases: 37 controls: 33	AN: 16.40 ± 2.33 CG: 16.51 ± 3.79	F = 100%	DSM-IV EDI-2	AN	PTQ
Opwis et al. 2017 [87]	cross-sectional	general population: 295	F: 30.23 ± 8.94 M: 30.76 ± 9.14	F: 69% (205)	EDE-Q	Eating pathology	RS-8
Wang and Borders 2017 [88]	cross-sectional	general population: 116	24.8 ± 5.35	M: 59.5% (69)	EAT-26	Eating pathology	Items modified from the Rumination About Interpersonal Offences Scale
Wang et al. 2017 [89]	cross-sectional	cases: 237	47.9 ± 10	F: 70% (167)	DSM-IV-TR EDE	BED	RRS
Dondzillo et al. 2018 [90]	cross-sectional	general population: 73	18.59 ± 1.28	F = 100%	DEBQ	AN	RRS-ED
Van Durme et al. 2018 [91]	cross-sectional	general population: 397	14.02	F: 62.7% (249)	EDI-II	BN	FEEL-KJ
Birmachu et al. 2019 [92]	cross-sectional	general population: 300	22.99 ± 6.91	F: 63.6% (190)	EDE-Q	Eating pathology	RRQ
Fresnics et al. 2019 [93]	cross-sectional	general population: 190	19.3 ± 1.10	F: 84% (160)	EDE-Q	Eating pathology	RRS
Hernando et al. 2019 [94]	case-control	cases: 25 controls: 25	16.6 ± 2.24 vs. 19.08 ± 0.64	F = 100%	-	AN, BN, OSFED	RSQ
Smith et al. 2019 [95]	cross-sectional	undergraduate students: 263	20.3 ± 3.68	F: 74.9% (197)	EDDS	Eating pathology	RRS
Branley-Bell and Talbot 2020 [96]	cross-sectional	general population: 129	9.27 ± 8.99	F: 93.8% (121)	Self-reported ED	Eating pathology	RRS-ED

Note: AN: Anorexia Nervosa; BN: Bulimia Nervosa; CG: control group; OSFED: Other Specified Feeding or Eating Disorders; ICD: International Classification of Diseases; DSM: Diagnostic and Statistical Manual of Mental Disorders; EAT-26: Eating Attitudes: Test EDE: Eating Disorder Examination; EDDS: Eating Disorder Diagnostic Scale; EDI-2: Eating Disorder Inventory; RSQ: Response Styles Questionnaire; BES: Binge Eating Scale; EDE-Q: Eating Disorders Examination Questionnaire; EDS-5: Eating Disturbance Scale; BARQ: Behavioural Anger Response Questionnaire; RRS: Ruminative Response Scale; RRS-ED: Ruminative Response Scale for Eating Disorders; MINI: Mini International Neuropsychiatric Interview; AFCI: Adult Emotion-Focused Coping Inventory; ChEAT: Children’s Eating Attitudes Test; CRSQ: Children’s Response Style Questionnaire; CERQ: Cognitive Emotion Regulation Questionnaire; EDE-Q: Eating Disorder Examination Questionnaire; RS-8: Rumination–Suppression-8 Scale; CERTS: Cambridge Exeter Repetitive Thought Scale; DEBQ: Dutch Eating Behaviour Questionnaire; FEEL-KJ: Fragebogen zur Erhebung der Emotionsregulation bei Kindern und Jugendlich; RRQ: Rumination–reflection Questionnaire.

**Table 3:** Moderator analyses: subtypes of ED symptoms, presence vs. absence of diagnosis of EDs, worry–rumination and age.

	ES	β Point Estimate	SE	CI	*p*
**Subtypes of ED symptoms**					**0.003**
AN	1.35		0.20	0.96–1.74	<0.001
BN	0.75		0.11	0.54–0.97	<0.001
BED	0.50		0.15	0.20–0.80	0.001
**Presence vs. absence of diagnosis of EDs**					**0.004**
Clinical samples	1.14		0.13	0.87–1.40	<0.001
Healthy controls from the general population	0.69		0.08	0.54–0.85	<0.001
**Repetitive Negative Thinking**					0.118
Worry	1.04		0.14	0.76–1.32	<0.001
Rumination	0.79		0.08	0.64–0.94	<0.001
**Age**		−0.0108	0.009	−0.03–0.01	0.26

Note: ES = Effect Size; SE = Standard Error; CI = Confidence Interval; AN = Anorexia Nervosa; BN = Bulimia Nervosa; BED: Binge Eating Disorder.

## Data Availability

Not applicable.

## Supplementary Materials

The following are available online at https://www.mdpi.com/article/10.3390/jcm10112448/s1, Table S1: Quality Assessment for Case-Control Studies using the Newcastle-Ottawa Scale, Table S2: Quality Assessment for Cross Sectional Studies using the Newcastle-Ottawa Scale.
