# Supplementary material for: Repetitive Negative Thinking and Eating Disorders: A Meta-Analysis of the Role of Worry and Rumination
Source: J Clin Med. 2021 May 31;10(11):2448. doi: 10.3390/jcm10112448 (PMC8198834; doi:10.3390/jcm10112448)
Supplement: Supplementary file 1 [file jcm-10-02448-s001.zip › jcm-1184455-supplementary.pdf]

Journal of *Clinical Medicine*

MDPI

Table S1. Quality Assessment for Case-Control Studies using the Newcastle-Ottawa Scale.

	Selection				Comparability		Exposure				
					Comparability cases-controls		Ascertainment of exposure				
Study	Study design (independent validation of cases)	Representativeness of the sample[a]	Selection of controls (community controls)	Definition of controls (no history of disease)	Matched by first factor[b]	Matched by second factor[c]	Secure record	Structured interview[d]	Same method of ascertainment for cases and controls	Response rate same for cases and controls	Total score (10)
Napolitano, & Himes, 2011 [31]			+	+				+	+	+	5/10
Sassaroli et al., 2005 [32]		+	+		+	+				+	5/10
Sternheim et al., 2012 [33]		+	+	+	+	+			+	+	7/10
Kollei et al., 2012 [58]		+	+	+	+	+			+	+	7/10
Naumann et al., 2015 [59]		+	+	+	+	+			+	+	7/10
Sapuppo et al., 2018 [60]			+	+	+	+			+	+	6/10
Troop & Treasure, 1997 [61]			+	+	+	+			+		5/10
Rawal et al., 2010 (study 2) [63]		+	+	+	+	+			+	+	7/10
Crino et al., 2019 [68]		+	+	+	+	+			+	+	7/10
Naumann et al., 2016 [85]	+	+	+	+					+	+	6/10
Seidel et al., 2016 [86]		+	+	+	+	+			+		6/10
Hernando et al., 2019 [94]		+	+	+	+	+			+	+	7/10

Note: + = indicates that the investigated characteristic is present in the study; a = Criteria: (1) during defined period; (2) during defined area; (3) all cases in a defined group; (4) appropriate sample (random or consecutive); b = age; c = sex; d = blinding case-control status.

Table S2. Quality Assessment for Cross Sectional Studies using the Newcastle-Ottawa Scale.

	Selection					Comparability	Outcome				
				Ascertainment of the exposure			Assessment of the outcome				
**Study**	**Representa-tiveness of the sample**	**Sample size**	**Non-respondents (satisfactory response rate is)**	**Validated measurement tool**	**Non-validated tool but it is described**	**Based on the study design and analysis**	**Independent blind assessment**	**Record linkage**	**Self report**	**Statistical test**	**Total score (10)**
González et al., 2017 [57]	+	+	+	++					+	+	7/10
Cowdrey & Park, 2012 [62]	+	+	+	++					+	+	7/10
Rawal et al., 2010 Study 1 [63]	+	+	+	++					+	+	7/10
Wang & Borders, 2018 study 1 [64]	+	+	+	++					+	+	7/10
Wang & Borders, 2018 study 2 [64]	+	+	+	++					+	+	7/10
Mason & Lewis, 2017 [65]	+	+	+	++					+	+	7/10
Startup et al., 2013 [66]	+	+		++					+	+	6/10
Zarychta et al., 2017 [67]	+	+	+	++					+	+	7/10
Hartmann et al., 2019 [69]	+	+	+		+				+	+	6/10
Connolly et al., 2007 [70]	+	+	+	++					+	+	7/10
Nolen-Hoeksema et al., 2007 [71]	+	+	+	++					+	+	7/10
Harrell et al., 2008 [72]	+	+	+	++					+	+	7/10
Selby et al., 2008 [73]	+	+	+	++		+			+	+	8/10

Aldao & Nolen-Hoeksema, 2010 [74]	+	+	+	++				+	+	7/10
Holm-Denoma & Hankin, 2010 [75]	+	+	+	++				+	+	7/10
Verplanken & Tangelder, 2011 [76]	+	+	+	++				+	+	7/10
Gordon et al., 2012 [77]	+	+	+	++		+		+	+	8/10
Kelly et al., 2012 [78]	+	+	+	++				+	+	7/10
Hilt et al., 2013 [79]	+	+	+	++				+	+	7/10
Svaldi & Naumann, 2014 [80]	+	+	+	++				+	+	7/10
Mason & Lewis, 2015 [81]	+	+	+	++				+	+	7/10
Breithaupt et al., 2016 [82]	+	+	+	++				+	+	7/10
Jungmann et al., 2016 [83]	+	+	+	++				+	+	7/10
Maraldo et al., 2016 [84]	+	+	+	++				+	+	7/10
Opwis et al., 2017 [87]	+	+	+	++				+	+	7/10
Wang & Borders, 2017 [88]		+	+		+			+	+	5/10
Wang et al., 2017 [89]	+	+	+	++				+	+	7/10
Dondzillo et al., 2018 [90]	+	+	+	++		+		+	+	8/10
Van Durme et al., 2018 [91]	+	+	+	++				+	+	7/10

Birmachu et al., 2019 [92]	+	+	+	++		+	+	7/10
Fresnics et al., 2019 [93]	+	+	+	++		+	+	7/10
Smith et al., 2019 [95]	+	+	+	++		+	+	7/10
Branley-Bell & Talbot, 2020 [96]	+	+	+	+		+	+	6/10

Note: + = indicates that the investigated characteristic is present in the study. In the section "Selection: Ascertainment of the exposure" two + must be assigned if the study use validated measurement tool, while one + mist be assigned in case of non-validated measurement tool, but the tool is available or described
